# Body image and attitudes toward cosmetic surgeries among female college students in the United Arab Emirates: A cross-sectional study

**DOI:** 10.1016/j.heliyon.2025.e42027

**Published:** 2025-01-17

**Authors:** Ahmad M. Al-Bashaireh, Yousef Aljawarneh, Osama Alkouri, Foad Alzoughool, Amal Alhefeiti, Amnah Aldhuhoori, Amnah Al Kaabi, Aseilah Alkaabi, Fawzia Alnaqbi

**Affiliations:** a Department of Nursing, Faculty of Health Sciences, Higher Colleges of Technology, P. O. Box: 1626, Abu Dhabi, United Arab Emirates; b Faculty of Nursing, Yarmouk University, P. O. Box: 566, Irbid, Zip code: 2116, Jordan; c Department of Medical Laboratory Sciences, Faculty of Applied Medical Sciences, The Hashemite University, Zarqa, P. O. Box 330127, Zarqa, 13133, Jordan

**Keywords:** *Cosmetic surgery*, *Body image*, *Female students*, *United Arab Emirates*

## Abstract

**Background:**

Due to the continuously increasing popularity of cosmetic surgeries, the influence of physical and facial beauty is pervasive worldwide. It encourages females to seek to meet specific beauty standards and not be content with their natural features. This study aimed to assess knowledge and motivation toward cosmetic surgeries, determine the prevalence of cosmetic surgeries, and examine the relationships between body image and attitude toward cosmetic surgeries among female college students.

**Methods:**

This study utilized a cross-sectional study design. Data were collected using Demographic Data Questionnaires, Cosmetic-related Questionnaire, the Body Appreciation Scale (BAS), and the Attitude Toward Cosmetic Surgery Scale (ACSS).

**Results:**

A total of 783 participants were enrolled in this study. The mean age of the participants was 20.95 years (SD = 2.149). The result showed that 47.6 % of participants considered cosmetic surgery necessary. While awareness of the potential side effects associated with cosmetic surgery varied among participants, most respondents acknowledged the existence of side effects (55.3 %). The most recognized type of cosmetic surgery among the respondents was filler (70.1 %). The primary motivation for cosmetic surgery was to enhance their appearance (67.9 %). On the other hand, the reasons for not having cosmetic surgery included high costs (49.4 %). Most participants (65.3 %) had no experience with any form of cosmetic surgery. Botox and filler were the most common types experienced by 5 % and 13.5 % of participants, respectively. Participants reported high body image scores (4.09 ± 0.95) and attitudes toward cosmetic surgeries (3.89 ± 1.42). The study found no significant correlation between body image and attitude toward the acceptance of cosmetic surgery (r = - 0.016, p < 0.697). However, a group who received cosmetic surgery was found to have a lower significant score of body image but a higher significant level for score of attitudes toward cosmetic surgery than the group who did not receive cosmetic surgeries.

**Conclusion:**

This study found that female college students showed varying levels of awareness regarding the side effects associated with cosmetic procedures. The most recognized type of cosmetic surgery among respondents was fillers, and the primary motivation for seeking such procedures was the desire to enhance appearance, while cost is the main reason for not seeking cosmetic surgeries. Furthermore, a study found those who received cosmetic surgeries reported a lower level of body image but a higher level of acceptance of cosmetic surgeries. The study emphasizes the need for future research endeavors to delve deeper into this subject matter using more robust research designs and methodologies. Moreover, a culturally tailored educational campaign is needed to enhance students' capacity for making informed decisions and instill a conscientious approach to personal health and aesthetic considerations.

## 1 Introduction

In light of the continuously increasing popularity of cosmetic surgeries, the influence of physical and facial beauty is pervasive worldwide. In a way, it encourages females these days to meet specific standards of beauty and not be content with their natural features [1]. After Saudi Arabia and Egypt, the UAE is considered to have the third-highest number of clients undergoing cosmetic surgery in the Middle Eastern region in 2021, and most of them are female [2]. For many, the influence of beauty and perfection leads to cosmetic surgery as an easy and fast solution that is available at different prices and available to everyone [2]. The importance of beauty of self-worth, worry about social standing, and attractiveness, investment in appearance, media influence on body image, and positive attitudes towards social media influencers are all factors that have been accepted in cosmetic surgeries among college students in the United States [3].

Attitudes and beliefs towards cosmetic surgeries have both positive and negative impacts on female students, and the perception of cosmetic surgeries was found to be positively associated with body image investments such as the desire to feel better and increased self-esteem [3]. On the other hand, negative attitudes were reported, such as worrying about weight, physical comparison with others, watching extreme beauty shows, low self-esteem, and exposure to cosmetics [3]. Furthermore, media literacy interventions reduced females' positive attitudes about elective cosmetic surgeries, body dysmorphic disorder, and body dissatisfaction while also increasing self-esteem [[4], [5], [6], [7], [8]].

The media often portrays unattainable beauty standards, which contributes to feelings of inadequacy and a desire to fit into these artificially constructed norms [[7], [8], [9]]. As a result, college students may consider cosmetic surgery as a possible solution to resolve the perceived difference between their current body image and society's idea of beauty [8,9]. Moreover, Hammadi and El-Shereef (2017) conducted a study on knowledge, attitudes, and practices related to cosmetic surgeries among female students of the Faculty of Education at Taif University in Saudi Arabia. There were 220 students who responded to the surveys. Participants who had never heard of cosmetic surgeries before learned about them via the media, and a quarter of the female students had the greatest understanding of what cosmetic surgeries entailed [10].

Body image encompasses the complex psychological aspects of how one perceives and relates to their own body, involving self-perceptions and self-attitudes, which encompass thoughts, beliefs, emotions, and behaviors related to the body [11]. Aside from the progress in cosmetic surgery technology and increased affordability, other factors might influence women's perspectives on cosmetic surgery. One of these factors includes the perception of the body, called body image [12]. According to Patalay and Hardman (2019), it is emphasized that mental images are often constructed unconsciously through a fusion of sensory perceptions, ideas, and emotions, forming and reshaping over the course of one's life. As depicted in most of today's studies, females are the most concerned about their body image, especially college students [13]. During the dynamic phase of college, young females are engaged in self-exploration, social interactions, and exposure to a multitude of beauty standards. In this transformative period, body image emerges as a central pillar of their self-concept. Its significance resonates in how it shapes their self-esteem and emotional equilibrium [14].

Studies showed that young women expressed interest in cosmetic surgery more frequently than young males, further these studies found individuals undergoing cosmetic surgery were primarily motivated by dissatisfaction with their appearance, the desire to enhance their attractiveness, and concerns about social acceptance [3,[15], [16], [17], [18], [19]]. In 2023, a study by Alshami and Asaati found that most people, especially women, were content with their noses and didn't want cosmetic nose surgery. They thought it was expensive, this suggests that people's choices about cosmetic surgery depended on how they felt about their appearance and the cost [20]. Notably Alghamdi et al. (2023) found that older individuals, married, employed, possessed postgraduate degrees, or enjoyed a higher economic status tended to exhibit high levels of acceptance toward cosmetic interventions [21]. However, women who engaged in more unhealthy weight management behavior tended to express a greater interest in cosmetic surgery [18]. The study by Kasmaei et al. (2020) found the intention to get cosmetic surgery was predicted by body satisfaction, image, and attitudes [22]. In the study conducted by Amiri et al. (2021), several noteworthy findings emerged regarding cosmetic surgeries in the UAE [2]. Their research highlighted a burgeoning acceptance of these surgeries along with a conspicuous alignment with Western beauty standards among UAE residents. A positive relationship was found between participants' self-esteem and their inclination to undergo cosmetic surgery [23]. Women may choose to undergo cosmetic surgery for a variety of reasons, and it's important to recognize that each person's motivations are unique to their circumstances and preferences. There were limited studies about this topic within the UAE population; therefore, this study aims to assess knowledge and motivation toward cosmetic surgeries, determine the prevalence of cosmetic surgeries, and examine the relationships between body image and attitude toward cosmetic surgeries among female college students.

## 2 Methods

### 2.1 Research design and population

This study utilized a cross-sectional research design to aims to assess knowledge and motivation toward cosmetic surgeries, determine the prevalence of cosmetic surgeries, and examine the relationships between body image and attitude toward cosmetic surgeries among female college students. The target population in this study comprised undergraduate female students. The accessible population included all ungraduated female students studying at the Higher Colleges of Technology (HCT). The total number of students enrolled at HCT is 24,933, and of them, 17,728 are female students. The inclusion criteria in this study encompassed HCT female students who were willing to participate in this study. The sample size for this study was calculated *A priori* using G∗Power software (G∗Power 3.1.9.7). For a point biserial correlation test with a small effect size measuring the correlation between the dependent (attitude toward cosmetic surgeries) and independent variables (body image), with a power of 0.8 on a two-tailed significant level of α = 0.05, a minimum of 779 participants were required in this study. To accommodate a 10 % drop-out rate, a total of 857 participants were needed in this study. The participants in this study were recruited using a convenience sampling technique.

### 2.2 Data collection procedure

The data collection conducted between 26 and 02–02024 and 10-04-2024 after the ethical approval by Research Ethics Integrity Committee (REIC) at the HCT (REIC2024-CAP05). The study measuring tools were converted to an online survey using Microsoft Form. Potential participants were approached face-to-face to invite them to participate in the study. Those who agreed to participate were provided with informed consent that included the aim of the research, the method and means of collecting data, the instrument used to collect data, and the risks and benefits associated with participation in the stud. Eligible participants were provided with the link to complete it on their own time. The expected time to complete the study survey was 7–10 min.

### 2.3 Research setting

This study was conducted at the Higher College of Technology, which is the largest academic institution in the United Arab Emirates, boasting 16 campuses spread across 7 emirates. The total student enrolment at HCT is 24,933, with 17,728 female undergraduate students. There were Abu Dhabi Women's Campus, Al Ain Women's Campus, Dubai Women's Campus, Fujairah Women's Campus, Madinat Zayed Women's Campuses, Ras Al Khaimah Women's Campus, Ruwais Women's Campus, and Sharjah Women's Campus.

### 2.4 Ethical considerations

The study received ethical approval from the HCT Research Ethics and Integrity Committee (REIC2024-CAP05). Data collection had not begun until ethical approval was granted. Participation was anonymous, and researchers did not ask for the participant's name, email, or phone number. Participation in the study was entirely voluntary, and participants were able to give informed consent as to whether they would participate in the study or not. Informed consent was required from the participants to confirm their willingness to participate in the study. There were no direct benefits from participation in this study, and participation did not entail any academic, physical, emotional, or psychological harm. All participants were treated equally. The collected data were confidential and used for research purposes only. Likewise, the collected data were secured in a password-protected device that was accessed only by the study Principal Investigator.

### 2.5 Data collection instruments

**Demographics Questionnaire:** This questionnaire was developed specifically for this study by the study researchers. The section had four questions about general sociodemographic questions, such as age, marital status, years of study, and campus.

**Cosmetic-related Questionnaire (knowledge, motivation, and practice):** This questionnaire was developed specifically for this study by the study researchers. The section had six cosmetic-related questions: necessity of cosmetic surgery for individual, main reasons for cosmetic surgery, main reasons for not having cosmetic surgery awareness about types of cosmetic surgery, awareness of side effects of cosmetic surgeries, and final question about if participants had experience of receiving cosmetic surgery.

**Body Image:** Body image was measured using the 10-item Body Appreciation Scale-2 (BAS-2). The scale was developed by Tylka & Wood-Barcalow in 2015 to assess the individual appreciation of body appearance [24]. The scale contained 10 items, and for each item, the following response scale was used: 1 = Never, 2 = Seldom, 3 = Sometimes, 4 = Often, 5 = Always. The scores were computed following the continuous scoring protocol by computing the mean scores across the items, with higher scores indicating a better (higher) appreciation of body image. The mean scores were then converted into two categories following the categorical scoring protocol: low body image appreciation (1–2.5) and high body image appreciation (2.6–5.0). The results showed satisfactory internal consistency reliabilities for the questionnaire our study with Cronbach's α of 0.94.

**Attitude Toward Cosmetic Surgery:** Attitude toward cosmetic surgeries was measured using the 15-item Acceptance of Cosmetic Surgery Scale (ACSS). The scale was developed by Henderson-King & Henderson-King in 2005 to measure participants' attitudes towards cosmetic surgery [25]. For each item, the following response scale was used: 1 = Strongly disagree, 2 = Disagree somewhat, 3 = Disagree a little, 4 = Neutral, 5 = Agree a little, 6 = Agree somewhat, 7 = Strongly agree. The scores were computed following the continuous scoring protocol by computing the mean scores across the items, with higher scores indicating a positive attitude toward acceptance of cosmetic surgeries. The scores were then converted into two categories following the categorical scoring protocol: negative attitude toward acceptance of cosmetic surgeries (1–3.5) and positive attitude toward acceptance of cosmetic surgeries (3.6–7.0). The ACSS was found to be reliable, with research findings supported in both Western and Chinese studies [26]. The results showed satisfactory internal consistency reliabilities for the questionnaire with Cronbach's α of 0. 0.93.

### 2.6 Data analysis

All data were analyzed using the SPSS software (IBM SPSS Statistics, Version 29, 2023). Descriptive statistics were calculated in terms of counts and percentages for categorical variables and means and Standard Deviation (SD) for continuous variables. Several statistical tests were employed, including Pearson's or Spearman's correlation test to examine the correlation between body image and attitudes toward cosmetic surgery. Independent *t*-test was used to compare mean scores of body image and attitudes toward cosmetic surgery in a group who had experience in receiving cosmetic surgery and a group who did not receive this experience. A p-value of <0.05 was considered statistically significant in all the analyses.

## 3 Results

The study received responses from a total of 848 participants. 26 declined to participate, and 39 were excluded for not meeting the eligibility criteria, resulting in a final sample size of 783 participants. The mean age of the participants was 20.95 years (SD = 2.15), with ages ranging from 17 to 34 years. Most of the participants were singles (89.1 %). The participants were distributed almost equally across the four years of the study as follows, 164 in year 1 (20.95 %), 212 in year 2 (27.1 %), 158 in year 3 (20.2 %), and 249 in year 4 (31.8 %). Additionally, a significant proportion of the participants were from the Fujairah Women's Campus (n = 509, 65 %) (Table 1).

**Table 1:** Participants’ demographics characteristics (N = 783).

Variable		n (%) or Mean ± SD
Age		20.95 ± 2.149
Marital Status	Single	698 (89.1 %)
	Married	69 (8.8 %)
	Divorced	8 (1.0 %)
	Widowed	6 (0.8 %)
	Separated	2 (0.3 %)
Year Study	Year1	164 (20.9 %)
	Year2	212 (27.1 %)
	Year3	158 (20.2 %)
	Year4	249 (31.8 %)
Campus	Abu Dhabi Women's	58 (7.4 %)
	Al Ain Women's	34 (4.3 %)
	Dubai Women's	48 (6.1 %)
	Fujairah Women's	509 (65.0 %)
	Madinat Zayed Women's	7 (0.9 %)
	Ras Al Khaimah Women's	39 (5 %)
	Ruwais Women's	10 (1.3 %)
	Sharjah Women's	78 (10 %)

Table 2 shows participants’ cosmetic-related characteristics such as necessity for cosmetic surgery, main reasons for cosmetic surgery, main reasons for not having cosmetic surgery awareness about types of cosmetic surgery, awareness of side effects of cosmetic surgeries, and if participants had experience of receiving cosmetic surgery. Concerning the necessity of cosmetic surgery, out of the 783 respondents, 373 (47.6 %) considered cosmetic surgery to be necessary, highlighting a significant proportion that sees it as essential rather than optional. Conversely, 410 participants (52.4 %) did not view cosmetic surgery as a necessity. Awareness of the potential side effects associated with cosmetic surgery varied among participants, reflecting differing levels of knowledge. The majority of respondents (n = 433, 55.3 %), acknowledged the existence of side effects. However, 144 respondents (18.4 %) were not aware of any side effects, indicating a significant knowledge gap that could impact decision-making regarding cosmetic surgery. Additionally, 206 participants (26.3 %) were unsure, reflecting uncertainty or insufficient information about the potential negative outcomes.

**Table 2:** Participants’ cosmetics-related characteristics (knowledge, motivation, and practice) (N = 783).

Variable		n (%)
Necessity towards cosmetic surgery	No: Not necessary	410 (52.4 %)
	Yes: Necessary	373 (47.6 %)
Awareness of side effects towards cosmetic surgery	No: Not aware of side effects	144 (18.4 %)
	Not sure	206 (26.3 %)
	Yes: aware of side effects	433 (55.3 %)
Knowledge of cosmetic surgery types (who answer yes for this item)	Botox	464 (59.3 %)
	Filler	549 (70.1 %)
	Cosmetic Breast Surgery	338 (43.2 %)
	Liposuction	304 (38.8 %)
	Reconstructive Surgery	199 (25.4 %)
	Rhinoplasty	270 (34.5 %)
	Others	224 (28.6 %)
Reasons for having cosmetic surgery (who answer yes for this item)	For beauty	532 (67.9 %)
	Increase self-esteem	516 (65.9 %)
	For reconstructive reasons	283 (36.1 %)
	Medical reasons	529 (67.6 %)
	Others	224 (28.6 %)
	No reasons	225 (28.7 %)
Reasons for not having cosmetic surgery (who answer yes for this item)	Not necessary	310 (39.6 %)
	High cost	387 (49.4 %)
	Adverse events	242 (30.9 %)
	Religion reasons	345 (44.1 %)
	All the reasons	374 (47.8 %)
Personal experience of receiving cosmetic surgery	None	511 (65.3 %)
	Botox	39 (5.0 %)
	Filler	106 (13.5 %)
	Cosmetic Breast Surgery	23 (2.9 %)
	Liposuction	27 (3.4 %)
	Reconstructive Surgery	13 (1.7 %)
	Rhinoplasty	13 (1.7 %)
	Others	51 (6.5 %)

The survey assessed participants' familiarity with different types of cosmetic surgeries, displaying a wide range of awareness. The most recognized type of cosmetic surgery among the respondents was filler, with 549 participants (70.1 %) indicating awareness. Botox followed closely, known to 464 respondents (59.3 %) (Table 2). The motivations behind choosing to undergo cosmetic surgery varied widely among the participants, reflecting diverse personal and societal influences. The primary reason reported for beauty, with 532 respondents (67.9 %) indicating this motivation. The second most common reason was medical reasons, identified by 529 participants (67.6 %), suggesting that many view cosmetic surgery as a tool not only for enhancing appearance but also for addressing medical issues, potentially increasing the quality of life. Increased self-esteem was also a significant factor, with 516 individuals (65.9 %) acknowledging it (Table 2). Concerning reasons for not having cosmetic surgery, the most prevalent concern was that it was not necessary, a view held by 310 participants (39.6 %). This perspective likely reflects a contentment with natural appearance or a belief that cosmetic surgery is an unnecessary luxury rather than a necessity. High cost was another significant deterrent, cited by 387 respondents (49.4 %), indicating that financial barriers play a crucial role in the decision-making process around cosmetic surgery. Adverse events, or potential side effects and complications, were a concern for 242 individuals (30.9 %), highlighting awareness of the risks involved with surgical interventions. This suggests a well-informed subset of the population that weighs health risks against the potential cosmetic benefits (Table 2).

A significant majority of the survey participants, 511 out of 783 (65.3 %), reported having no experience with any form of cosmetic surgery. Among the surveyed participants, non-invasive procedures like Botox and filler are the most common, experienced by 39 (5.0 %) and 106 (13.5 %) participants, respectively, likely due to their less invasive nature and shorter recovery times. Surgical procedures such as cosmetic breast surgery, liposuction, reconstructive surgery, and rhinoplasty are less common, with experiences reported by 23 (2.9 %), 27 (3.4 %), 13 (1.7 %), and 13 (1.7 %) participants, respectively (Table 2)

Table 3 shows the mean scores of body image and acceptance of cosmetic surgery. The mean score on the Body Appreciation Scale (BAS) was 4.09 (SD = 0.95), indicating a high level of body appreciation among participants. This suggests that, on average, participants tended to have positive attitudes toward their own bodies, with relatively low variability in their responses. Concerning categories of body image, the results showed that, out of a total of 783 participants, only 46, representing 5.9 % of the sample, have a low level of body appreciation. Conversely, most of the sample, comprising 737 individuals (94.1 %) reported a high level of body appreciation (Tablet 4). The mean score of Acceptance of Cosmetic Surgery Scale (ACSS) was 3.89 (SD = 1.42) indicating a high level of acceptance of cosmetic surgery among participants (Table 3). Concerning categories of acceptance of cosmetic surgery, the results showed that 265 participants (33.8 %) showed a negative attitude toward acceptance of cosmetic surgery, while a larger portion of participants (n = 518, 66.2 %) exhibited a positive attitude toward acceptance of cosmetic surgery (Table 4).

**Table 3:** Average scores of Body Appreciation Scale (BAS) and the Acceptance of Cosmetic Surgery Scale (ACSS) (N = 783).

Scales	Mean ± SD
Body Appreciation Scale (BAS)	4.09 ± 0.95
Acceptance of Cosmetic Surgery Scale (ACSS)	3.89 ± 1.42

**Table 4:** Levels of body appreciation scale (BAS) and the acceptance of cosmetic surgery scale (ACSS) (N = 783).

Scales		n (%)
Body Appreciation Scale (BAS)	Low level	46 (5.9 %)
	High level	737 (94.1 %)
Acceptance of Cosmetic Surgery Scale (ACSS)	Negative	265 (33.8 %)
	Positive	518 (66.2 %)

This study's findings revealed no significant correlation between body image and attitude toward cosmetic surgery (r = −0.014, p = 0.697). Table 5 compares mean scores of body image and attitude toward cosmetic surgery between the group who received and the group who did not receive cosmetic surgery. Compared with the group who had not received cosmetic surgery, the group who received cosmetic surgery was found to have a lower significant score of body image (3.66 ± 1.11 vs. 4.32 ± 0.76, t = 8.846, *p* < 0.001) but a higher significant level for score of attitudes toward cosmetic surgery (4.47 ± 1.32 vs. 3.58 ± 1,38, t = −8.762, *p* < 0.001).

**Table 5:** Comparison of body image and attitude toward cosmetic surgery between groups who received and group who were not received cosmetic surgery (N = 783).

Outcome variables	Independent variables	Mean ± SD	df	T value	P value
	**Experience of receiving cosmetic surgery (number: 511 no, 272 yes)**				
Body image	No	4.32 ± 0.76	781	8.846	<0.001∗∗∗
	Yes	3.66 ± 1.11			
Attitude toward cosmetic surgery	No	3.58 ± 1.38		−8.726	<0.001∗∗∗
	Yes	4.47 ± 1.32			

## 4 Discussion

The present study found a significant discrepancy in perceptions regarding the necessity of cosmetic surgery with 47.6 % of the participants expressing the belief that cosmetic surgery is essential, highlighting considerable support for its perceived necessity. While this majority may appear modest, it underscores a usual lack of emphasis on the importance of cosmetic surgery. Regarding knowledge about side effects, the study found that among female students undergoing cosmetic surgery, (55.3 %) experienced side effects, indicating an informed understanding of associated risks. However, a significant knowledge gap was evident, with (18.4 %) unaware of any side effects and (26.3 %) lacking sufficient information about negative consequences. In support of our findings, a study conducted in Saudi Arabia among female college students revealed notable differences in acceptance levels and awareness of potential side effects. The study found that 29 % of female college students expressed consideration of future cosmetic surgery, while 31.1 % of the students expressed their willingness to undergo cosmetic surgery if side effects were absent. On the other hand, 67.4 % of the participants in the Saudi Arabian study rejected cosmetic surgeries, even if offered for free, and 46.8 % expressed unwillingness to undergo any cosmetic surgery [21]. Therefore, the findings of our study underscore shifting levels of acceptance and reluctance towards cosmetic surgery, likely influenced by social factors.

Regarding knowledge towards cosmetic surgery types, the present study revealed varied familiarities with various types of cosmetic surgery among participants. The most recognized type was Filler, followed by Botox These non-invasive procedures are likely more familiar due to their prevalence in media and relatively simple, low-risk profiles. Comparatively, a study conducted by Sayegh et al. (2024) and Bin Rubaian et al. (2024) in the Kingdom of Saudi Arabia found that noninvasive cosmetic procedures such as laser hair removal, fillers and Botox were the most common cosmetic procedures, particularly among younger age groups, which suggests a global trend towards noninvasive cosmetic enhancements [27,28]. This parallels our findings and highlights a general shift in cosmetic preferences towards treatments that are perceived as safer and require minimal recovery time. On the other hand, our study showed relatively lower awareness and knowledge about more invasive procedures like reconstructive surgery (25.4 %) and rhinoplasty (34.5 %). This contrasts with findings from a study conducted in Saudi Arabia that reported a significant interest in rhinoplasty among female high school students, possibly influenced by cultural and media portrayals of beauty standards that favor facial symmetry and specific nose shapes [29]. This indicates that cultural and regional differences may influence the awareness and acceptance of certain types of cosmetic surgeries. Furthermore, Zhang et al. (2024) discovered that female college students who reported a low-rated physical appearance, lived closer to the job market, or predicted a greater salary were more likely to undergo cosmetic surgery [8]. This finding suggests that professional demands and societal expectations can significantly affect individuals' knowledge and attitudes toward cosmetic surgeries, a trend that was less pronounced in our study group but relevant in contexts where appearance is closely tied to career success.

The present study also assessed the reasons for having cosmetic surgery. The findings showed that among female college students, the primary motivation for undergoing cosmetic surgery is the desire to enhance their appearance. The majority of participants (67.9 %) identified enhancing beauty as the primary motivation for undergoing cosmetic surgery. A comparison with a study conducted among students in Saudi Arabia reveals intriguing differences. While only 26.3 % of Saudi Arabian students considered cosmetic surgery for beauty reasons, while 48.8 % - the highest proportion in their study - may undergo cosmetic interventions due to medical reasons [21]. Additionally, our study identified medical reasons as the second most common motivation (67 %) among participants. A study was conducted in South Korea in 2020 to explore the perception of cosmetic surgery and associated side effects among female college students. It found that nearly half of participants believed people are drawn to cosmetic surgery for various reasons, including a desperate desire for a change in appearance, believe in the absence of negative side effects, and increased self-esteem [14]. This is consistent with our finding, where the primary motivation for undergoing cosmetic surgery is often linked to enhancing beauty, addressing medical reasons, and enhancing self-esteem. These comparisons highlight the diverse factors that influence the decision to undergo cosmetic surgeries across different cultural contexts.

The present study also assessed the reasons for not having cosmetic surgery. The results revealed that fear and religious beliefs were the main reasons people were choosing not to have cosmetic surgery. In our study, high costs emerged as a significant concern, with 49.4 % of participants citing it as a barrier. This contrasted sharply with just 12.8 % reported in a similar study conducted among students in KSA. Similarly, religious reasons influenced 44.1 % of our participants, slightly surpassing the 35.8 % noted in the Saudi Arabian context, indicating a strong moral or ethical stance against cosmetic alterations. Concerns about adverse events were also more prevalent in our sample (30.9 %) than in Saudi Arabia (21.5 %). Particularly, a significant 47.8 % of our respondents considered all these factors together when deciding against cosmetic surgery, significantly more than the mere 1.5 % in the Saudi study, suggesting a more comprehensive approach to decision-making among our participants [21].

Regarding personal experience of receiving cosmetic surgery, the findings of the present study align with studies conducted across different cultures and countries. A study by Ateq et al. (2024) found a higher acceptance of cosmetic surgeries in Saudi participants with body dysmorphic disorder. Further, a study by Aladwan et al. (2023) reported that enhancing look and attractiveness was one of the significant reasons in Jordanians who underwent cosmetic surgeries [19]. Sterian et al. (2023) reported that self-esteem and body image in Romanian participants were the strongest predictors for cosmetic surgeries [16]. Furthermore, studies from China and South Korea found that dissatisfaction with body area and physical appearance increased college students' likelihood of cosmetic surgeries [8,30]. This echoes the findings of several studies across different cultures and countries, which likely captured similar dynamics among its participants. These studies help us learn more about how many female college students get cosmetic surgery, why they do it, and how they feel about it. They show that it's essential to understand these things from different cultural perspectives [8,30].

The present study assessed the participant's body image and acceptance of cosmetic surgery about the motivation toward cosmetic surgery. Comparing the descriptive statistics on body image and acceptance of cosmetic surgery between the present study and Al Ghadeer et al. (2021) offers valuable insights into the perceptions and attitudes of participants from the Eastern Province of Saudi Arabia [23]. Our study, comprising 783 participants, reported a body image score of 4.09 (SD = 0.95) and a mean Attitude toward cosmetic surgery score of 3.89 (SD = 1.42), indicating high levels of body appreciation and acceptance of cosmetic surgery. Similarly, Al Ghadeer et al.'s research, involving 1008 participants, found a notably high body admiration rate (74.2 %) and moderate acceptability towards cosmetic surgery (55.4 %). Both studies underscore prevalent positive attitudes toward body image and cosmetic surgery acceptance among participants from the Eastern Province of Saudi Arabia. While our study provided specific mean scores for BAS and ACSS, Al Ghadeer et al., focused more on the prevalence rates of body admiration and acceptability towards cosmetic surgery [23]. Also, Cypriot nursing students reported a similar moderate acceptability toward cosmetic surgery [31]. These complementary findings contribute to a comprehensive understanding of the cultural and psychological factors influencing attitudes towards cosmetic surgery acceptance in the region, informing future research and interventions aimed at promoting positive body image and informed decision-making regarding cosmetic surgeries.

The present study showed no significant correlation between body image and attitude toward cosmetic surgery. Another study conducted by Al Ghadeer and his colleagues reported a significant inverse correlation between appropriation of the body and acceptance of cosmetic surgery [23]. Additionally, correlational analysis in a study by Gillen and her colleagues revealed an association between higher body appreciation and lower interest in cosmetic surgery [18]. This difference suggests that the relationship between body image and attitude toward cosmetic surgery may vary across different populations or may be influenced by various factors.

### 4.1 Strengths and limitations

There are several strengths and limitations in this study. Regarding the strengths, although the study utilized the convenience sampling technique to recruit participants, which may affect the generalizability of the findings, the large sample size is considered one of the study's strengths by approaching broader aspects of the target population. Additionally, this study stands out as the only research conducted in the United Arab Emirates on the attitude and perception toward cosmetic surgery among female college students. In contrast, the study's limitations include its cross-sectional design, which makes it difficult to establish cause and effect and interpret relationships between variables. Generalizing our findings to other populations is warranted since this study was focused on the female college student. This study did not screen subjects for psychological disorders that may be associated with cosmetic surgeries; future restudies are recommended to screen for these disorders to have a more comprehensive view of this phenomenon. Furthermore, using self-administered measuring tools is one of the limitations, potentially leading to recall bias and responses based on the respondents' perception, who may not be able to assess themselves or report on their own experience accurately. Moreover, religion, religious groups, and levels of religiosity and spirituality were only partially explored among the subjects. Studying these factors in future research would provide further insight into how these factors influence attitudes toward body image and cosmetic surgeries.

## 5 Conclusion

This study found that female college students showed varying levels of awareness regarding the side effects associated with cosmetic procedures. The most recognized type of cosmetic surgery among respondents was fillers, and the primary motivation for seeking such procedures was the desire to enhance appearance, while cost is the main reason for not seeking cosmetic surgeries. Furthermore, a study found those who received cosmetic surgeries reported a lower level of body image but a higher level of acceptance of cosmetic surgeries. The study emphasizes the need for future research endeavors to delve deeper into this subject matter using more robust research designs and methodologies. Moreover, a culturally tailored educational campaign is needed to enhance students' capacity for making informed decisions and instill a conscientious approach to personal health and aesthetic considerations.

### 5.1 Recommendations

In light of the findings from this study, which demonstrated a pronounced interest in cosmetic surgeries among female college students and a concomitant significant knowledge gap about the risks of more invasive surgeries, it is recommended that specialized educational interventions be implemented. To bridge these gaps, the introduction of comprehensive awareness campaigns is advised, specifically targeting the detailed understanding of both invasive and non-invasive cosmetic surgeries. Moreover, it is proposed that these educational programs be tailored to fit the unique needs and interests identified within the student cohort, as evidenced by the variability in awareness and perceptions regarding various cosmetic surgeries. These initiatives are intended to provide the students with thorough knowledge and insights, thereby enhancing their capacity for making informed decisions about cosmetic enhancements and instilling a conscientious approach towards personal health and aesthetic considerations. In addition, this study recommends future research studies to explore this phenomenon in detail with more robust research designs and methodologies.

## CRediT authorship contribution statement

**Ahmad M. Al-Bashaireh:** Writing – review & editing, Writing – original draft, Formal analysis, Conceptualization. **Yousef Aljawarneh:** Writing – review & editing, Writing – original draft, Methodology. **Osama Alkouri:** Writing – review & editing, Writing – original draft, Visualization, Methodology. **Foad Alzoughool:** Writing – original draft, Visualization, Investigation. **Amal Alhefeiti:** Writing – original draft, Validation, Data curation. **Amnah Aldhuhoori:** Methodology, Investigation, Data curation. **Amnah Al Kaabi:** Writing – original draft, Investigation. **Aseilah Alkaabi:** Writing – original draft, Investigation. **Fawzia Alnaqbi:** Writing – original draft, Investigation.

## Ethical approval statements

• The study was approved by the Research Ethics and Integrity Committee at the Higher Colleges of Technology on February 23, 2024 (Reference Number: REIC2024-CAP05).
• The study was performed per the ethical standards laid down in the 1964 Declaration of Helsinki and its later amendments.
• Informed consent was obtained from all individual participants included in the study.

## Data availability statement

The data that supports the findings of this study are available in the supplementary material of this article.

## Funding

The authors declare that they have no known competing financial interests or personal relationships that could have appeared to influence the work reported in this paper.

## Declaration of competing interest

The authors declare that they have no known competing financial interests or personal relationships that could have appeared to influence the work reported in this paper.
