# Supplementary material for: Body image and attitudes toward cosmetic surgeries among female college students in the United Arab Emirates: A cross-sectional study
Source: Heliyon. 2025 Jan 17;11(2):e42027. doi: 10.1016/j.heliyon.2025.e42027 (PMC11791236; doi:10.1016/j.heliyon.2025.e42027)
Supplement: Multimedia component 1 [file mmc1.docx]

**Demographic Data Questionnaire and Cosmetic-related Questionnaire (knowledge, motivation, and practice)**

1. What is your age in years? (……………….)
2. Marital status:

- Married
- Single
- Divorced
- Widowed
- Separated
- Prefer not to tell

1. Year of study?

- Year 1
- Year 2
- Year 3
- Year 4

1. Which campus?

- Abu Dhabi Women’s Campus
- Al Ain Women’s Campus
- Dubai Women’s Campus
- Fujairah Women’s Campus
- Madinat Zayed Women’s Campus
- Ras Al Khaimah Women’s Campus
- Ruwais Women’s Campus
- Sharjah Women’s Campus

1. Is cosmetic surgery always necessary for individuals to feel their best?

- Yes
- No

1. Do all cosmetic surgeries have potential side effects?

- Yes
- No
- I don’t know

1. What type/s of cosmetic surgery are aware of: Please select all that apply:

- Botox
- Filler
- Cosmetic breast surgery
- Liposuction
- Reconstructive surgery
- Rhinoplasty
- Others

1. What do you think are the main reasons for cosmetic surgery? please select all that apply:

- For beauty
- Increase self-esteem
- For reconstructive reasons
- Medical reasons
- Others
- No reasons for cosmetic surgery

1. What do you think are the main reasons for not having cosmetic surgery? please select all that apply:

- Not necessary
- High cost
- Adverse events
- Religion reasons
- All the reasons

1. Do you have any previous experience of receiving cosmetic surgery?

- Yes
- No
- If your answer is yes:
- Botox
- Filler
- Cosmetic breast surgery
- Liposuction
- Reconstructive surgery
- Rhinoplasty
- Others

**10-item Body Appreciation Scale-2 (BAS-2)**

| **10-item Body Appreciation Scale** | **Never** | **Seldom** | **Sometimes** | **Often** | **Always** |
| --- | --- | --- | --- | --- | --- |
| 1. I respect my body. | 1 | 2 | 3 | 4 | 5 |
| 1. I feel good about my body. | 1 | 2 | 3 | 4 | 5 |
| 1. I feel that my body has at least some good qualities. | 1 | 2 | 3 | 4 | 5 |
| 1. I take a positive attitude towards my body. | 1 | 2 | 3 | 4 | 5 |
| 1. I am attentive to my body’s needs. | 1 | 2 | 3 | 4 | 5 |
| 1. I feel love for my body. | 1 | 2 | 3 | 4 | 5 |
| 1. I appreciate the different and unique characteristics of my body. | 1 | 2 | 3 | 4 | 5 |
| 1. My behavior reveals my positive attitude toward my body; for example, I hold my head high and smile. | 1 | 2 | 3 | 4 | 5 |
| 1. I am comfortable in my body. | 1 | 2 | 3 | 4 | 5 |
| 1. I feel like I am beautiful even if I am different from media images of attractive people (e.g., models, actresses/actors). | 1 | 2 | 3 | 4 | 5 |

**15-item Acceptance of Cosmetic Surgery Scale (ACSS)**

| **15-item Acceptance of Cosmetic Surgery Scale** | **Strongly disagree** | **Disagree Somewhat** | **Disagree a little** | **Neutral** | **Agree a little** | **Agree Somewhat** | **Strongly agree** |
| --- | --- | --- | --- | --- | --- | --- | --- |
| 1. It makes sense to have minor cosmetic surgery rather than spending years feeling bad about the way you look. | 1 | 2 | 3 | 4 | 5 | 6 | 7 |
| 2. Cosmetic surgery is a good thing because it can help people feel better about themselves. | 1 | 2 | 3 | 4 | 5 | 6 | 7 |
| 3. In the future, I could end up having some kind of cosmetic surgery. | 1 | 2 | 3 | 4 | 5 | 6 | 7 |
| 4. People who are very unhappy with their physical appearance should consider cosmetic surgery as one option. | 1 | 2 | 3 | 4 | 5 | 6 | 7 |
| 5. If cosmetic surgery can make someone happier with the way they look, then they should try it. | 1 | 2 | 3 | 4 | 5 | 6 | 7 |
| 6. If I could have a surgical procedure done for free I would consider trying cosmetic surgery. | 1 | 2 | 3 | 4 | 5 | 6 | 7 |
| 7. If I knew there would be no negative side effects or pain, I would like to try cosmetic surgery. | 1 | 2 | 3 | 4 | 5 | 6 | 7 |
| 8. I have sometimes thought about having cosmetic surgery. | 1 | 2 | 3 | 4 | 5 | 6 | 7 |
| 9. I would never have any kind of plastic surgery. | 1 | 2 | 3 | 4 | 5 | 6 | 7 |
| 10. I would think about having cosmetic surgery in order to keep looking young. | 1 | 2 | 3 | 4 | 5 | 6 | 7 |
| 11. If it would benefit my career I would think about having plastic surgery. | 1 | 2 | 3 | 4 | 5 | 6 | 7 |
| 12. Cosmetic surgery can be a big benefit to people’s self-image. | 1 | 2 | 3 | 4 | 5 | 6 | 7 |
| 13. If a simple cosmetic surgery procedure would make me more attractive to others, I would think about trying it. | 1 | 2 | 3 | 4 | 5 | 6 | 7 |
| 14. I would seriously consider having cosmetic surgery if my friend Or family thought it was a good idea. | 1 | 2 | 3 | 4 | 5 | 6 | 7 |
| 15. I would seriously consider having cosmetic surgery if I thought my friends or family would find me more attractive. | 1 | 2 | 3 | 4 | 5 | 6 | 7 |
